# High Sensitivity Refractometer Based on TiO_2_-Coated Adiabatic Tapered Optical Fiber via ALD Technology

**DOI:** 10.3390/s16081295

**Published:** 2016-08-15

**Authors:** Shan Zhu, Fufei Pang, Sujuan Huang, Fang Zou, Qiang Guo, Jianxiang Wen, Tingyun Wang

**Affiliations:** 1 Key Laboratory of Specialty Fiber Optics and Optical Access Networks, Shanghai University, 149 Yanchang Road, Shanghai 200072, China; ss_zhu@shu.edu.cn (S.Z.); sjhuang@shu.edu.cn (S.H.); zf-198166@sohu.com (F.Z.); qguo@staff.shu.edu.cn (Q.G.); wenjx@shu.edu.cn (J.W.); tywang@shu.edu.cn (T.W.); 2 Hebei GEO University, 136 East Huaian Road, Shijiazhuang 050031, China

**Keywords:** refractometer, adiabatic tapered optical fiber, titanium dioxide nanofilm, atomic layer deposition

## Abstract

Atomic layer deposition (ALD) technology is introduced to fabricate a high sensitivity refractometer based on an adiabatic tapered optical fiber. Different thicknesses of titanium dioxide (TiO_2_) nanofilm were coated around the tapered fiber precisely and uniformly under different deposition cycles. Attributed to the higher refractive index of the TiO_2_ nanofilm compared to that of silica, an asymmetric Fabry–Perot (F-P) resonator could be constructed along the fiber taper. The central wavelength of the F-P resonator could be controlled by adjusting the thickness of the TiO_2_ nanofilm. Such a F-P resonator is sensitive to changes in the surrounding refractive index (SRI), which is utilized to realize a high sensitivity refractometer. The refractometer developed by depositing 50.9-nm-thickness TiO_2_ on the tapered fiber shows SRI sensitivity as high as 7096 nm/RIU in the SRI range of 1.3373–1.3500. Due to TiO_2_’s advantages of high refractive index, lack of toxicity, and good biocompatibility, this refractometer is expected to have wide applications in the biochemical sensing field.

## 1. Introduction

Optical fiber refractometers are of great importance to the biochemical sensing field due to the advantages of high sensitivity, compact size, and anti-electromagnetic interference. For most of the fiber-based refractometers, the response of the sensor to changes in surrounding refractive index (SRI) is nonlinear, in which the sensitivity is much higher for an SRI close to 1.45 (which is the fiber material refractive index), but lower for an SRI close to 1.33 [1,2]. In order to improve SRI sensitivity in the vicinity of an SRI of 1.33 (which is the environment refractive index of the most of biosensors), nanofilm-coated optical fibers with metallic materials, semiconductor materials or high refractive index dielectric materials have been widely investigated [3,4,5,6,7,8,9,10,11,12,13]. One strategy is the deposition of high refractive index nanofilm on fibers which exhibit a mode resonant feature. These fibers include long period gratings (LPGs) fiber [3,4,5], double cladding fiber (DCF) [6], and cascading single-mode–multi-mode–single-mode (SMS) fiber [7]. It has been demonstrated that the sensitivity of these SRI sensors can be enhanced by mode reorganization behavior. However, since the SRI sensitivity can only be improved significantly in the transition region, the enhancement is limited. Hence, the schemes based on fibers without mode resonant feature are developed, including adiabatic tapered optical fiber, D-shaped optical fiber, cladding-removed multimode optical fiber, etc. Depending on the properties of the nanofilm, these fiber-optic refractometers without mode resonance work based on either surface Plasmon resonance (SPR) [8,9] or lossy modes resonance (LMR) [10,11,12,13]. For SPR-based refractometers, the real part of the permittivity of the coated nanofilm is negative and higher in magnitude than both its own imaginary part and the permittivity of the material surrounding the nanofilm. In this case, the evanescent wave in the fiber is coupled to the surface Plasmon polariton. For LMR-based refractometers, the fiber is coated by high refractive index-absorbing nanofilm, whose real part of permittivity is positive and higher in magnitude than both its own imaginary part and the permittivity of the material surrounding the nanofilm, such as indium tin oxide (ITO) [10,11], titanium dioxide (TiO_2_) [12], aluminum oxide (Al_2_O_3_) [13], and [PAH-PAA]_X_ polymeric thin film [14]. Light guided in these structures is coupled to the lossy mode in the nanofilm. LMR has the advantage that it can be generated using a wide variety of materials in a wide spectral range.

The common technologies used to deposit nanofilms on optical fiber include dip-coating method [15], self-assembly method [12,14], sputtering technique [10,11], etc. However, precise control of film thickness cannot be achieved by dip-coating method. Self-assembly method requires the precise control of external parameters (pressure, humidity, temperature, etc.) to produce homogeneous and repetitive nanofilm. For sputtering technique, it is difficult to form a uniform nanofilm around the fiber’s cylindrical surface. Recently, atomic layer deposition (ALD) technology is considered to be one of the most promising nanofilm deposition techniques in optical fiber fields [16]. ALD technology is a conformal deposition process through sequential, self-limiting surface reactions. Compared with conventional fiber coating technologies, ALD technology has special advantages, including accurate thickness control, good conformity for complex shapes, excellent step coverage, and good uniformity and adhesion. Many studies have shown that ALD is very suitable for coating a nanofilm on the rod shape of an optical fiber to realize functional fiber components [13,17,18,19].

Adiabatic tapered optical fiber sensors have been studied in previous work by coating Al_2_O_3_ via ALD technology. It was demonstrated that SRI sensitivity can be improved by coating with nanofilms with higher refractive indices [13]. TiO_2_ has a higher refractive index than Al_2_O_3_, with advantages such as lack of toxicity and good biocompatibility, making it more suitable for biochemical sensing. In this paper, we have experimentally demonstrated that TiO_2_ can be coated on tapered optical fiber by using ALD technology for high sensitivity refractometers. Theoretical analysis is also conducted based on ray optics to explain the interference resonance inside the fiber. The optical field confined in the tapered fiber leaks out into the nanofilm, and multiple beam interference occurs, which can be regarded as a thin film Fabry–Perot (F-P) resonator. Its resonant transmission spectrum depends strongly on the SRI variation, which allows the detection of SRI with high sensitivity. The refractometer developed by depositing 50.9-nm-thickness TiO_2_ on the tapered fiber shows an SRI sensitivity of 7096 nm/RIU in the SRI range of 1.3373–1.3500.

## 2. Theory

**Figure 1:**
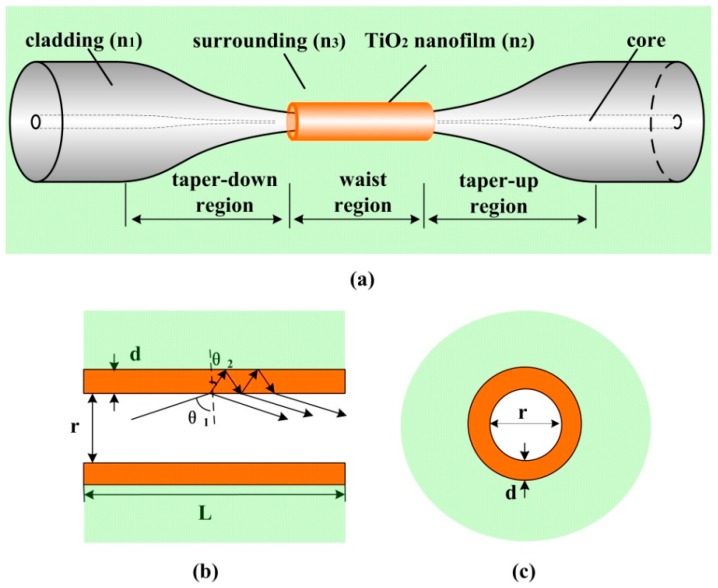
Schematic diagram of (**a**) the tapered fiber coated with TiO_2_ nanofilm, and the coated region in the (**b**) axial and (**c**) radial directions.

To simulate a sensor based on an adiabatic tapered optical fiber with a high refractive index nanofilm as shown in Figure 1, a theoretical model was developed, and the applicability of this method has been proven successfully in [13] for Al_2_O_3_-coated tapered optical fiber. For the adiabatic tapered fiber with a uniform waist, the transmission loss is almost negligible because of the weak energy of the excited high-order modes, and only fundamental mode is considered to propagate in the taper waist section [20]. Therefore, the theoretical model of the tapered fiber sensor can be simplified to be a nanofilm-coated cylindrical waveguide with infinite surrounding, where r refers to diameter, L refers to length, d refers to nanofilm thickness, $n_{3}$ refers to SRI, as shown in Figure 1b,c. Since the nanofilm refractive index is higher than that of the fiber taper, light waves cannot be confined in the fiber by total internal reflection (TIR). As depicted in Figure 1b, when the light strikes on the taper–nanofilm interface with grazing incidence, the light will be partially reflected, and then be completely reflected at the nanofilm–surrounding interface. As a result, multiple beam interference occurs along the tapered fiber, just like a thin film F-P interferometer. The transmission will present a periodic interference spectrum.

If the propagation light wave in the fiber taper is not polarized, the combination of the reflected power in s and p polarization should be taken into account. The light path presents a zigzag ray trajectory due to multiple reflections on the taper-nanofilm interface. The general expression for the transmission is [21]

(1) $$P={\left[\frac{1}{2}{\left|F_{s}\left(\lambda \right)\right|}^{2}+\frac{1}{2}{\left|F_{p}\left(\lambda \right)\right|}^{2}\right]}^{N}$$

$F_{s}$ and $F_{p}$ can be described as [22]

(2) $$F_{s}=\frac{r_{s1}+r_{s2}\exp\left(i\sigma \right)}{1+r_{s1}r_{s2}\exp\left(i\sigma \right)}$$

(3) $$F_{p}=\frac{r_{p1}+r_{p2}\exp\left(i\sigma \right)}{1+r_{p1}r_{p2}\exp\left(i\sigma \right)}$$

where $r_{s1}$ and $r_{p1}$ are the Fresnel coefficients for s and p polarization of the taper–nanofilm interface, and $r_{s2}$ and $r_{p2}$ are the Fresnel coefficients for s and p polarization of the nanofilm–surrounding interface, respectively. $\sigma =4\pi n_{2}d\cos\theta_{2}\lambda^{-1}$ is the phase delay induced by optical path difference, $n_{2}$ is the nanofilm refractive index, $\theta_{2}$ is the incidence angle on the nanofilm–surrounding interface. If the nanofilm is lossy, its refractive index becomes a complex one. $N=L/\left(r\tan\theta_{1}\right)$ is the number of reflections at the taper–nanofilm interface, and $\theta_{1}$ is the incidence angle on the taper–nanofilm interface. For the fundamental mode, can be expressed by $\theta_{1}=\arcsin\left(\beta_{HE11}/\left(k_{0}n_{1}\right)\right)$, where $k_{0}=2\pi /\lambda$, $\beta_{HE11}$ is the propagation constant of the fundamental mode, and $n_{1}$ is the cladding refractive index.

Since each incident angle corresponds to a fixed wavelength for the fundamental mode, the transmission presents interference dips at different wavelengths, as follows [23]:

(4) $$\lambda_{m}=\frac{4\pi n_{2}d\cos\theta_{2}}{\phi +2m\pi }$$

where ϕ is the phase delay induced by the Goos–Hänchen shift due to the TIR on the nanofilm–surrounding interface, $\phi =2\arctan\left(\sqrt{2\Delta \cos\theta_{2}^{-2}-1}\right)$ is for s polarization, $\phi =2\arctan\left(n_{2}^{2}n_{3}^{-2}\sqrt{2\Delta \cos\theta_{2}^{-2}-1}\right)$ is for p polarization, and $\Delta =\frac{1}{2}\frac{n_{2}^{2}-n_{3}^{2}}{n_{2}^{2}}$. It is obvious that the dips will shift as a function of the nanofilm thickenss, the nanofilm refractive index, and the SRI from Equation (4). When the nanofilm thickenss and refractive index are defined, the SRI sensing can be realized by monitoring the shifts of the dips.

**Figure 2:**
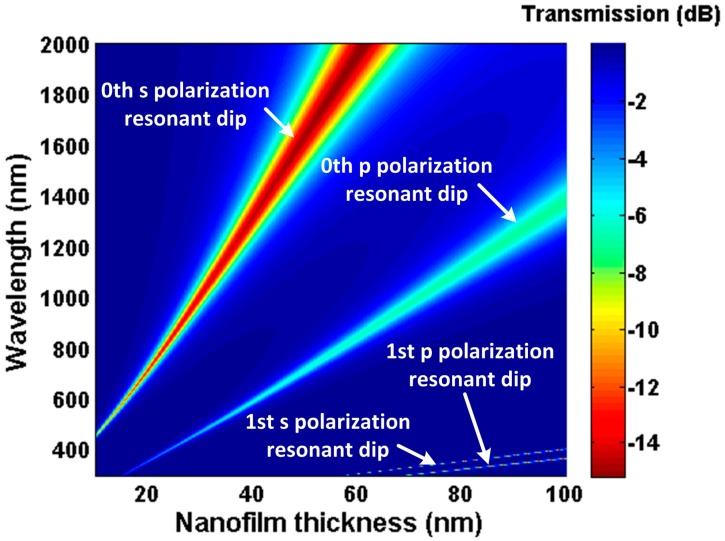
Transmission spectra as a function of the TiO_2_ film thickness in water (1.33).

**Figure 3:**
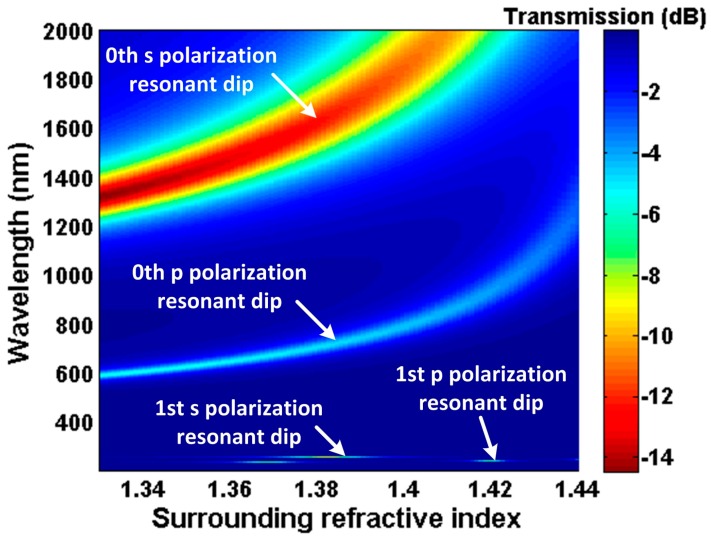
Transmission spectra as a function of surrounding refractive index (SRI) with d = 40 nm.

Simulation results using the above model are presented in Figure 2 and Figure 3. In the simulation, material dispersion of refractive index for the TiO_2_ nanofilm was referred from [24] (n_TiO__2_ ≈ 2.278 @ 1550 nm), and the imaginary part was set as 0.005. The waist diameter and length are 25 µm and 7 mm, respectively. In Figure 2, there are multiple resonant dips generated by the asymmetric F-P resonator. The mth resonant pairs correspond to different polarization transmission, which has been demonstrated in [13]. These resonant spectra present red shift and bandwidth broadening with the increase of the nanofilm thickness, and the low order resonant spectrum shifts faster than the high order one. Attributed to the spectral dependency of the nanofilm, the resonant wavelength can be tuned from the visible to the near-infrared wavelength band. For a definite nanofilm material, it is possible to tune the resonant dip conveniently by changing the nanofilm thickness.

As shown in Figure 3, we observe that the resonant wavelengths shift to longer wavelength as the SRI increases. According to Equation (4), when the nanofilm refractive index and thickness are defined, the variation of SRI causes $r_{s2}$ and $r_{p2}$ of the nanofilm–surrounding interface to change. As a result, the phase delay induced by the Goos–Hänchen shift changes, which leads to the shift of the transmission interference spectrum. Moreover, it can be found that the wavelength shift of the 0th s polarization resonant dip is larger than others; namely, the sensitivity of the 0th s polarization resonant dip is highest [13]. Hence, we only focus on the 0th s polarization resonant dip in the following work. By monitoring the shift of the 0th s polarization resonant dip, the change of the SRI can be obtained. Additionally, in Figure 2, the 0th s polarization resonant dip sweeps the range 500 nm–2000 nm between thickness 10 nm and 60 nm. Therefore, the resonant wavelength position (working point) can be controlled by changing the nanofilm thickness.

Meanwhile, temperature stability of the refractometer was studied. The thermo-optic coefficient of the TiO_2_ nanofilm, the fiber cladding, and the water is −4.2 ± 0.7 × 10^−5^/°C [24], 7.8 × 10^−6^/°C [25], and −9 × 10^−5^/°C [26], respectively. These refractive indices have slight changes with the temperature fluctuation. According to Equation (4), we can calculate the sensitivity of the 0th resonant dip to temperature (d = 40 nm), which is 0.48 nm/°C for the s polarization resonant dip and 0.18 nm/°C for the p polarization resonant dip. When SRI changes by 0.002 RIU (the SRI step in the experiment), a resonant wavelength shift of 9.0 nm for the s polarization resonant dip and 3.6 nm for the p polarization resonant dip can be achieved. However, a change in temperature of 18.8 °C is required to obtain the same amount of shift. Therefore, a shift in temperature by 1 °C results in a change of the measured refractive index by 1 × 10^−4^ RIU.

## 3. Fabrication of Tapered Fiber Coated with TiO_2_ Nanofilm

### 3.1. Adiabatic Tapered Fiber Fabrication

In this work, we used a heat-pulling system with oxyhydrogen flame to fabricate the adiabatic tapered optical fiber. The taper waist diameter and the total length are approximately 25 μm and 16 mm, respectively. The insertion loss of the pulled fiber taper is less than −1 dB.

### 3.2. TiO_2_ Nanofilm Deposition

To deposit the nanofilm around the tapered fiber waist, ALD equipment (TFS 200, Beneq) was utilized. The tapered fibers were placed in a cylindrical reaction chamber whose diameter and height were 22 cm and 3 cm, respectively.

The reaction temperature was kept at 140 °C. The two precursors (TiCl_4_ and H_2_O) and N_2_ gas were valve-controlled and pulsed into the reaction chamber with the order of TiCl_4_–N_2_–H_2_O–N_2_. When TiCl_4_ is introduced, the first half-reaction proceeds as −OH + TiCl_4_ →−OTiCl_3_ + HCl. When all of the available OH-sites are reacted, the half-reaction stops. HCl gas is released as the by-product, and the surface changes from −OH to −TiCl groups. Then, the excess TiCl_4_ and the HCl are purged away by N_2_. When H_2_O is introduced, the second half-reaction proceeds as −TiCl + H_2_O →−TiOH + HCl. This half-reaction forms Ti–O bands, and the surface is −OH groups, which creates sites for the reaction of the next deposition cycle. The excess H_2_O and the HCl are purged away by N_2_. The monolayer TiO_2_ film was deposited on the surface of the taper waist in 7 s. Repeating the deposition cycle, the TiO_2_ film can be deposited layer by layer. Due to the self-limiting growth mechanism, the monolayer thickness is almost the same in each deposition cycle, and the thickness of the final TiO_2_ film can be precisely controlled through the number of deposition cycles.

**Figure 4:**
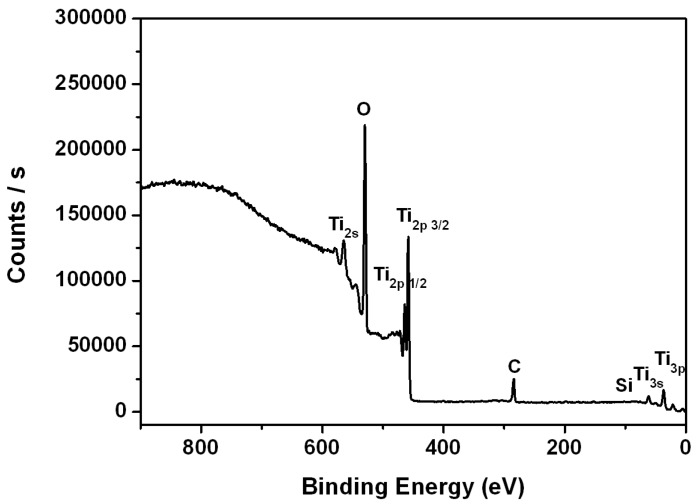
X-ray photoelectron spectroscopy (XPS) picture of optical fiber deposited with TiO_2_ nanofilm.

The TiO_2_ film deposited on the optical fibers was characterized using X-ray photoelectron spectroscopy (XPS) and scanning electron microscopy (SEM). Figure 4 shows the XPS results. The titanium and oxygen are registered respectively, which confirms that the TiO_2_ film has been successfully deposited.

**Figure 5:**
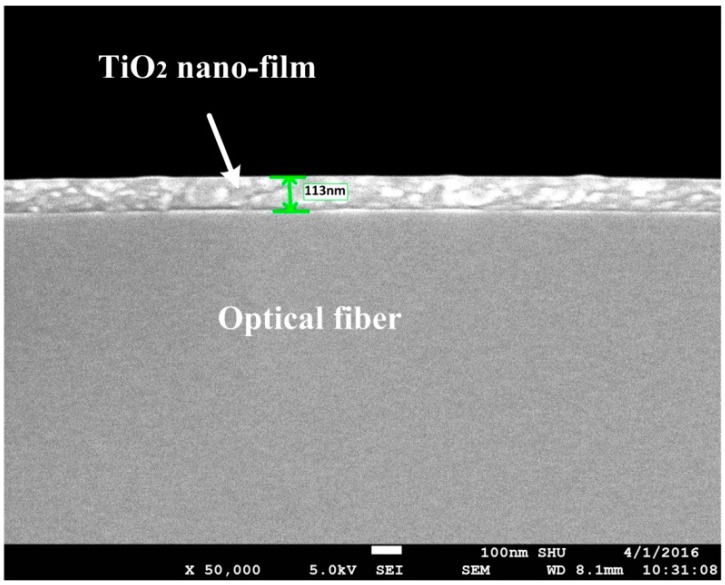
Scanning electron microscopy (SEM) picture of an optical fiber deposited with 3000 layers TiO_2_ nanofilm.

Figure 5 shows an SEM image of the cross section of an optical fiber deposited with 3000 layers of TiO_2_ film. In Figure 5, the thickness is approximately 113 nm, so the average thickness per layer is about 0.0377 nm.

## 4. Experiments and Results

**Figure 6:**
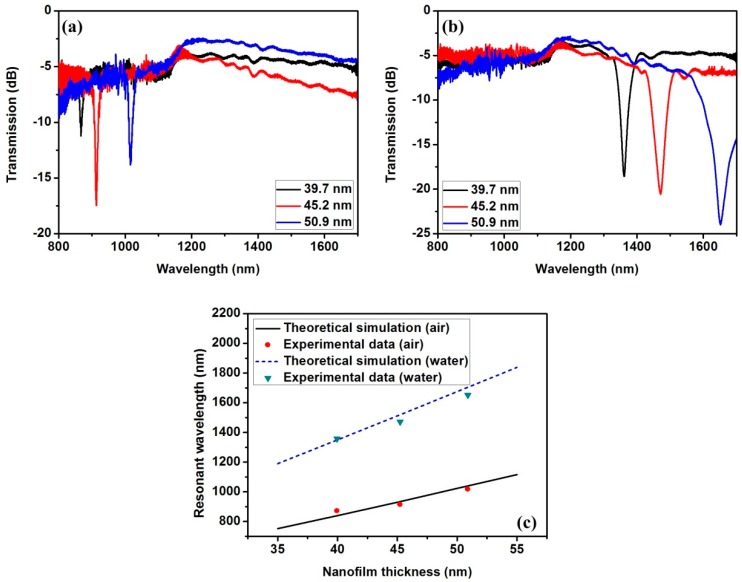
Transmission spectra obtained when the tapered fiber with TiO_2_ thicknesses of 39.7, 45.2, and 50.9 nm are immersed in (**a**) air and (**b**) deionized water; and (**c**) resonant wavelength as a function of the TiO_2_ film thickness.

With a broadband light source (NKT Photonics SuperK Compact, 500 nm–2400 nm) and two optical spectrum analyzers (AQ-6315A <600 nm–1700 nm>, AQ-6375 <1200 nm–2400 nm>), the transmission spectra of the tapered fiber with TiO_2_ film thickness of 39.7 nm, 45.2 nm, and 50.9 nm were measured in air and in deionized water, as shown in Figure 6a,b, respectively. We observe that the 0th resonant dips shift to the longer wavelength and the bandwidth of the resonant spectra are markedly broadened as the TiO_2_ film thickness increases, both in air and in deionized water. Moreover, for the TiO_2_ film with the same thickness, the resonant wavelengths exhibit a red shift with switching from air to deionized water, which indicates that the TiO_2_-coated adiabatic tapered fiber is sensitive to the change of SRI. Figure 6c shows that the shift of the resonant wavelength is linearly proportional to the nanofilm thickness. According to this relationship, we can set the deposition cycle to adjust the working point. The experimental results obtained in Figure 6c show good agreement with the theoretical results.

In the sensing experiment for SRI, the fiber taper sensor head was fixed on a glass microscope slide, and liquid samples with different refractive indices were dropped on the sensor head. A mixture of glycerol and deionized water in different ratios was used as the liquid sample. The SRI is from 1.3373 to 1.3500, which was certified by using an Abbe refractometer with a resolution of 0.0001. The temperature around the sensor head was maintained at approximately 20 °C in order to minimize the SRI/temperature cross sensitivity. In addition, the sensor head was cleaned by deionized water and dried after each sample measurement.

**Figure 7:**
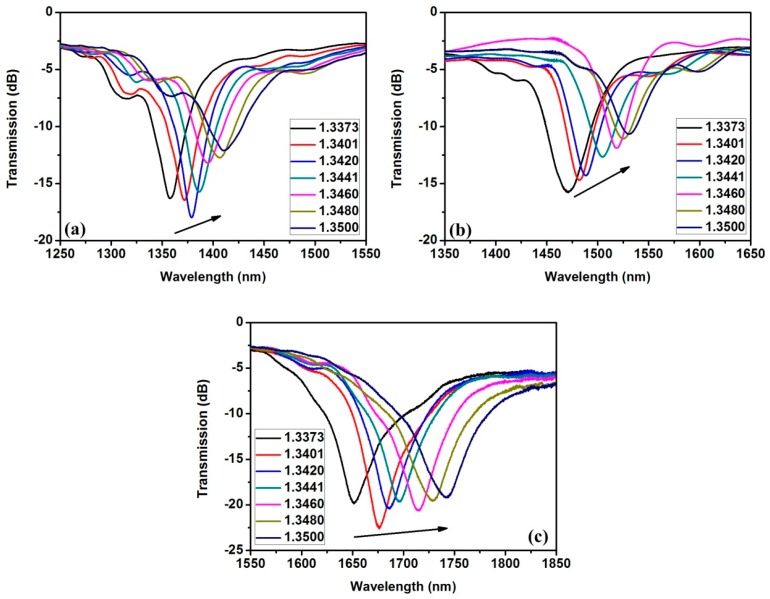
Transmission spectra shift with increasing SRI for different TiO_2_ film thickness values. (**a**) 39.7 nm; (**b**) 45.2 nm; (**c**) 50.9 nm.

Three sensors with TiO_2_ film thickness of 39.7 nm, 45.2 nm, and 50.9 nm were fabricated and investigated. The transmission spectra versus SRI are shown in Figure 7a–c. As the SRI increases, all of the transmission spectra shift toward the longer wavelength. According to Equation (4), this relationship results from the decrease in the phase delay induced by the Goos–Hänchen shift in the total internal reflection on the nanofilm–surrounding interface. In addition, the shift amount increases gradually with the increase of nanofilm thickness. The reason is that the incident angle for the same order resonant dip will increase to satisfy the phase interference condition, which will lead to an increase in the Goos–Hänchen phase delay change rate according to the TIR feature [23]. Hence, a greater amount of shift can be obtained with increasing nanofilm thickness. Namely, the shift amount increases as the resonant wavelength shifts to a longer wavelength.

**Figure 8:**
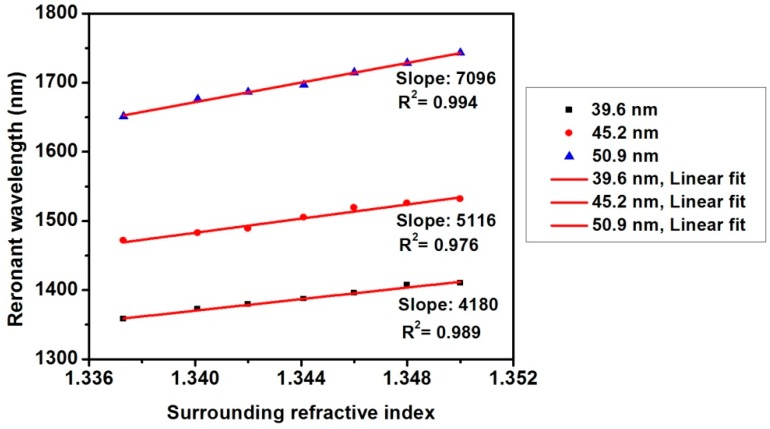
Relative resonant wavelength increases with increasing SRI for different TiO_2_ film thickness.

In Figure 8, we can also observe that the shift of the resonant wavelength is a linear function of the SRI. It shows that the sensitivity is enhanced for the sensor head with thicker nanofilms. The sensitivity of the tapered fiber with TiO_2_ film thickness of 39.7 nm, 45.2 nm, and 50.9 nm are 4180 nm/RIU, 5116 nm/RIU, and 7096 nm/RIU, respectively. Moreover, compared with the tapered fiber coated with Al_2_O_3_ nanofilm where the maximum sensitivity is 6008 nm/RIU [13], the sensitivity of the tapered fiber coated with TiO_2_ nanofilm has been improved, which results from the higher refractive index of TiO_2_. These results agree with previous discussion that either increasing nanofilm thickness or increasing nanofilm refractive index can improve the sensitivity for the same order resonant dip.

**Figure 9:**
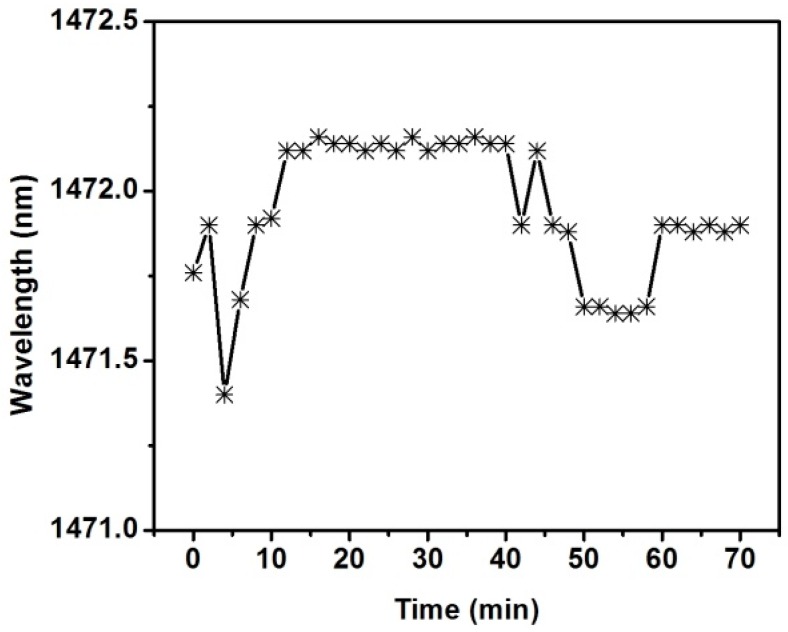
Resonant wavelength versus time.

The stability characteristic of the refractometer is also studied. The liquid sample with a refractive index of 1.3369 was dropped on the sensor head with a TiO_2_ film thickness of 45.2 nm. The transmission spectrum was acquired every 2 min. In order to minimize the effect of liquid sample evaporation on the experimental results during a 70-min test, the sensor head was sealed in a small plastic box. As shown in Figure 9, the resonant wavelength fluctuates very little over time, and the Root Mean Square (RMS) value of the fluctuation is about 0.2 nm. The change in refractive index corresponding to the measured wavelength fluctuation is 1.5 × 10^−4^. The result indicates that the refractometer has good stability.

## 5. Conclusions

In this paper, a TiO_2_-coated adiabatic tapered optical fiber was fabricated by ALD technology. The fabricated devices show a red shift of their resonant wavelength as the SRI increases, which enables their use for SRI sensing purposes. Since the phase delay induced by the Goos–Hänchen shift has a significant impact on the resonant phase condition for the 0th s polarization resonant dip, high sensitivity for SRI can be obtained. Based on the advantages of ALD technology, tunable thickness of the TiO_2_ nanofilm ensures precise control of the transmission spectrum. Moreover, the thickness also determines the sensitivity of the sensor. The experimental results demonstrate that the high sensitivity of 7096 nm/RIU in the range 1.3373–1.3500 can be obtained, which is better than Al_2_O_3_-coated adiabatic tapered fiber refractometers for the same order of resonant spectrum [13]. The results have proven that a higher refractive index nanofilm permits the achievement of improved sensitivity. In general, a refractometer based on a TiO_2_ nanofilm is more suitable for biochemical applications due to the advantages of TiO_2_, such as high refractive index, lack of toxicity, and good biocompatibility.
